# Endpoints in NASH Clinical Trials: Are We Blind in One Eye?

**DOI:** 10.3390/metabo14010040

**Published:** 2024-01-08

**Authors:** Amedeo Lonardo, Stefano Ballestri, Alessandro Mantovani, Giovanni Targher, Fernando Bril

**Affiliations:** 1 AOU—Modena—Ospedale Civile di Baggiovara, 41126 Modena, Italy; a.lonardo@libero.it; 2 ASL Modena—Ospedale di Pavullo, 41026 Pavullo nel Frignano, Italy; stefanoballestri78@gmail.com; 3 Section of Endocrinology and Diabetes, Department of Medicine, University of Verona, Piazzale Stefani, 37126 Verona, Italy; 4 Department of Medicine, University of Verona, 37126 Verona, Italy; giovanni.targher@univr.it; 5 Metabolic Diseases Research Unit, IRCCS Sacro Cuore—Don Calabria Hospital, 37024 Negrar di Valpolicella, Italy; 6 Department of Medicine, Heersink School of Medicine, University of Alabama at Birmingham (UAB), Birmingham, AL 35233, USA; fbril@uab.edu

**Keywords:** biomarkers, cardiovascular risk, liver biopsy, metabolic dysfunction, natural history

## Abstract

This narrative review aims to illustrate the notion that nonalcoholic steatohepatitis (NASH), recently renamed metabolic dysfunction-associated steatohepatitis (MASH), is a systemic metabolic disorder featuring both adverse hepatic and extrahepatic outcomes. In recent years, several NASH trials have failed to identify effective pharmacological treatments and, therefore, lifestyle changes are the cornerstone of therapy for NASH. with this context, we analyze the epidemiological burden of NASH and the possible pathogenetic factors involved. These include genetic factors, insulin resistance, lipotoxicity, immuno-thrombosis, oxidative stress, reprogramming of hepatic metabolism, and hypoxia, all of which eventually culminate in low-grade chronic inflammation and increased risk of fibrosis progression. The possible explanations underlying the failure of NASH trials are also accurately examined. We conclude that the high heterogeneity of NASH, resulting from variable genetic backgrounds, exposure, and responses to different metabolic stresses, susceptibility to hepatocyte lipotoxicity, and differences in repair-response, calls for personalized medicine approaches involving research on noninvasive biomarkers. Future NASH trials should aim at achieving a complete assessment of systemic determinants, modifiers, and correlates of NASH, thus adopting a more holistic and unbiased approach, notably including cardiovascular–kidney–metabolic outcomes, without restricting therapeutic perspectives to histological surrogates of liver-related outcomes alone.

## 1. Definitions, Burden, and Methods

### 1.1. NASH Belongs to the NAFLD Spectrum

Coined by Schaffner and Thaler in 1986, the “nonalcoholic fatty liver disease” (NAFLD) terminology covers the large spectrum of alcohol-like diseases in patients without significant alcohol consumption and other competing causes of hepatic steatosis [1]. In 1995, Lonardo et al. drew attention to metabolic dysfunction and cardiovascular disease being associated with “fatty liver syndrome” based on the clinical correlates of individuals with no excessive alcohol consumption and low prevalence of infection with hepatitis viruses who had hepatic steatosis on ultrasonography [2]. Four years later, three different teams of investigators independently reached the same conclusion, i.e., NAFLD is closely associated with metabolic syndrome and greater insulin resistance [3,4,5].

Recently, important advances regarding disease nomenclature for this common liver disease have resulted in the proposal of moving from a “negative” diagnostic criterion (i.e., nonalcoholic) to a “positive” one, i.e., “metabolic dysfunction-associated”. These proposals have led to NAFLD being renamed to metabolic dysfunction-associated fatty liver disease (MAFLD) in 2020 [6,7] and, to further avoid any residual stigmatization, to metabolic dysfunction-associated steatotic liver disease (MASLD) in 2023 [8]. A major difference between the MAFLD and MASLD definitions is that the latter suggests that the term steatohepatitis is an important pathophysiological concept that should be retained and is defined as metabolic dysfunction-associated steatohepatitis (MASH) [8].

NAFLD/MASLD is an umbrella definition spanning from uncomplicated hepatic steatosis (NAFL) to the more rapidly progressive nonalcoholic steatohepatitis (NASH), defined as concurrent steatosis with inflammation and hepatocyte injury, such as ballooning, with or without liver fibrosis [9]. However, advanced fibrosis, cirrhosis, and hepatocellular carcinoma occur only in a proportion of NAFLD individuals [10,11]. While the course of the liver disease is typically unpredictable in the individual NAFLD patient [12], the histologic severity of liver fibrosis will determine whether patients are more likely to be exposed to developing adverse liver outcomes, cardiovascular–kidney–metabolic complications, or certain types of extrahepatic malignancies [13,14,15]. The severity of liver fibrosis is also strongly associated with an increased risk of all-cause mortality and liver-related morbidity in patients with NASH [16], and NASH is the strongest predictor of fibrosis progression in NAFLD [17]. In their turn, metabolic risk factors strongly predict the development of NASH and liver fibrosis [18].

From this background of evidence, it has become increasingly evident that NAFLD, rather than being a disease exclusively confined to the liver, is instead a multisystem disease [19], whose manifestations and complications outreach—among the best characterized—the areas of glucose and lipid metabolism, extrahepatic cancers, chronic kidney disease, and cardiovascular disease (i.e., the latter representing the leading cause of mortality in people with NAFLD) [20,21,22,23].

### 1.2. NASH Is a Major Public Health Concern

Exacting a tremendous clinical and financial toll, NASH represents a public health concern calling for the prompt action of policy and clinical decision-makers [24].

The prevalence of NASH ranges from ~2% to 6.5% globally, with a nearly two-fold increase among high-risk individuals such as middle-aged individuals in the USA [25]. Of concern, NASH is increasing at an alarming pace worldwide. A recent study projected the occurrence by 2039 of ~18.7 million overall deaths, ~6.7 million cardiac-specific deaths, and ~1.7 million liver-specific deaths among patients with NASH in the USA, accounting for USD 1208.5 billion and USD 453.9 billion owing to estimated cumulative expenses for direct healthcare among NASH patients with and without obesity, respectively [26].

A fundamental feature in the epidemiological characteristics of NAFLD is sexual dimorphism in the development and progression of this liver disease, strongly modulated by reproductive status. In a meta-analysis of 54 observational studies, including 5428 individuals with NASH out of 62,239 with NAFLD (6444 with advanced liver fibrosis), Balakrishnan et al. [27] found that compared with men, women had a ~20% lower risk of NAFLD (pooled risk ratio (RR) 0.81; 95% CI 0.68–0.97), a similar risk of NASH (RR 1.00; 95% CI 0.88–1.14), and a 37% higher risk of advanced fibrosis (RR 1.37; 95% CI 1.12–1.68) in the general population. Interestingly, the risks of NASH and advanced fibrosis were substantially higher in women with ages of ≥50 years (RR 1.17; 95% CI 1.01–1.36 and RR 1.56; 95% CI 1.36–1.80, respectively), while these risks were attenuated among younger populations. However, sexual dimorphism is often a neglected feature in NASH clinical trials, despite the consideration of sex and reproductive status as a research priority in the NAFLD arena [28].

Although the prevalence of obesity in the USA is expected to reach a plateau, the global prevalence of NASH is predicted to keep rising until 2030, owing to the ongoing contribution of T2D in increasing the ratio of NASH to isolated steatosis within the next decade [29]. Worryingly, compared with previous decades, patients with NAFLD have arrived for medical observation with more severe liver disease and higher subclinical atherosclerosis burden [30]. This finding accounts for NASH being the most frequent indication for liver transplantation among elderly individuals [31].

### 1.3. At-Risk NASH

In 2020, Harrison et al. [32] defined “at-risk NASH” as NASH with an histological NAFLD activity score (NAS) ≥ 4 and fibrosis stage F ≥ 2 [24]. NAS is a histological composite score established by the NASH-CRN (Nonalcoholic Steatohepatitis Clinical Research Network) in 2005 [33]. It is obtained by summing up the histological scores for steatosis, hepatocellular ballooning, and lobular inflammation [33]. Identification of NASH trial endpoints to be used based on this histological scoring system was developed by the American Association for the Study of Liver Diseases (AASLD) and further developed by the multistakeholder Liver Forum [34].

The rationale for identifying patients with “at-risk NASH” is based on studies of the natural history of the disease, showing that NASH activity and fibrosis stage are the two strongest histological determinants of the long-term risk of severe liver-related outcomes [35]. In turn, the activity of liver inflammation in NASH, defined using the NAS score, is a definite driver of hepatic fibrogenesis [36]. At the same time, the stage of fibrosis faithfully mirrors the odds of disease progression toward cirrhosis and, therefore, the risk of liver-related clinical outcomes [36,37,38].

Based on recent analyses, ~9 million people in the USA are estimated to have “at-risk NASH,” and this condition is more prevalent in men than in women. Metabolic comorbidities, such as obesity, type 2 diabetes (T2D), metabolic syndrome, and insulin resistance are more common than in the general adult population, with T2D and increased waist circumference being independently associated with increasing odds of “at-risk NASH” [39].

While several noninvasive fibrosis biomarkers (such as blood-based tests and imaging techniques) are available for ruling out subjects with advanced fibrosis [40], identifying those with “at-risk NASH” is more challenging as these fibrosis biomarkers are not specifically designed to identify patients with “at-risk NASH” and, therefore, how to use such noninvasive biomarkers to this end is currently uncertain [41,42]. Interestingly, noninvasive fibrosis biomarkers are significantly associated with higher cardiovascular risk scores [43].

### 1.4. Criteria of Bibliographic Research

This narrative review is based on a critical analysis of pertinent published studies. To retrieve these studies, the following research strategy was adopted on 31 August 2023: ((NASH [Title/Abstract]) AND (trials [Title/Abstract])) AND (endpoints [Title/Abstract]). Additional studies were identified using the following keywords: “NASH resolution without fibrosis progression [Title/Abstract]”. Cross-references and articles from the authors’ archives were also consulted whenever indicated.

### 1.5. Aim

This perspective article specifically explores the epidemiological evidence regarding the association of NASH with T2D, obesity, and metabolic syndrome. Furthermore, we also highlight the distinctive systemic dysmetabolic-associated features of NASH pathogenesis. Next, the main reasons underlying the failure of NASH clinical trials are analyzed in parallel with the limitations of NASH histological endpoints. Finally, research perspectives are illustrated.

## 2. NASH as a Metabolic Disorder: Epidemiology and Mechanisms

### 2.1. NASH and T2D

NASH has a close and bidirectional association with T2D. Indeed, on the one hand, the prevalence of T2D among patients with NASH (estimated to be as high as 43.6%) is approximately five-fold higher than the prevalence of T2D in the general adult population (8.5%) [44]. On the other hand, the estimated global prevalence of NASH in people with T2D was 37.3% (95% CI 24.7–50.0%), with advanced fibrosis found in 17% (95% CI 7.2–34.8%) among those with NAFLD and T2D [45]. These figures support the notion that we could affect disease risk by acting on each component of this closed-circuit association. In other words, we could reduce the risk of developing T2D by treating NAFLD and improving NASH with many antihyperglycemic agents [46,47]. However, the precise mechanisms linking these two conditions are not entirely understood, and their close association may just be mediated by the underlying insulin resistance, a common ground of both conditions [48]. Addressing chronic hyperglycemia in T2D with glucose-lowering medications that are not insulin sensitizers and do not induce significant weight loss (e.g., insulin or DPP-4 inhibitors) has resulted in inconsistent results in NAFLD [49,50], downplaying the effect of chronic hyperglycemia per se on the relationship between T2D and the risk of NASH. The prevalence of NAFLD in people with type 1 diabetes (T1D) is highly variable and largely dependent on body mass index (BMI) and the diagnostic methodology used, being the highest in liver ultrasound-based studies, where the pooled prevalence was around 30% (ranging from ~10% to 65%), and the lowest in magnetic resonance imaging-based studies, where the pooled prevalence was about 10% (ranging from ~0% to 30%) [51]. In a cohort study of 530 Belgian adults with T1D, Mertens et al. recently reported that the prevalence of NAFLD appeared to mimic that of the general population once matched for BMI [52]. Despite the unclear link between hyperglycemia and the severity of liver disease in NAFLD, several “classically” considered glucose-lowering medications have shown significant efficacy in NAFLD and NASH. Among these, pioglitazone and glucagon-like peptide-1 (GLP-1) receptor agonists have the strongest evidence from randomized, controlled trials assessing liver histology as the primary outcome [53,54,55].

Pioglitazone has been shown to significantly reduce hepatic steatosis, achieve resolution of NASH, and delay fibrosis progression [54]. While data from randomized, controlled trials on its effects on liver fibrosis improvement have been inconsistent, a meta-analysis showed an overall reduction in the fibrosis stage with pioglitazone use [56]. As the liver benefits are similar in patients with and without T2D [57], it is postulated that the benefits of pioglitazone are secondary to improvement in insulin resistance.

Subcutaneous semaglutide is the GLP-1 receptor agonist with the strongest randomized controlled trial evidence showing a significant reduction in liver fat content and resolution of NASH [55]. Semaglutide also reduces fibrosis progression, although no significant improvement in fibrosis stages has been reported. In the absence of GLP-1 receptors in the human liver, it is postulated that the hepatic benefits of subcutaneous semaglutide are mostly linked to weight loss. Consistently, GLP-1 receptor agonists have shown similar benefits in patients with and without T2D.

Sodium–glucose cotransporter 2 (SGLT2) inhibitors are other glucose-lowering medications that have shown promising results in NAFLD. The evidence for SGLT2 inhibitors comes mainly from randomized, controlled trials using magnetic resonance imaging as the primary outcome [58,59]. While randomized, double-blind, controlled trials with histology-driven outcomes are lacking, some open-label studies have suggested improvement in several histological parameters [60]. While the debate is still open, it appears that hepatic benefits are also driven by the modest weight loss observed with these glucose-lowering agents. Like pioglitazone and GLP-1 receptor agonists, these drugs also seem to have similar effects in patients with and without T2D [61].

Overall, while several medications have been shown to tackle T2D and NASH simultaneously, it appears that they do so primarily by improving insulin resistance and/or producing weight loss, but not due to their glucose-lowering properties.

Box 1 summarizes the most important points in the link between NAFLD/NASH and T2D.

Box 1 **Epidemiological data highlighting a mutual and bidirectional association between NAFLD/NASH and T2D.**

 T2D aggravates the natural history of NAFLD, increasing the odds of progression to advanced fibrosis, cirrhosis, HCC, LT, and death [46].

 Persistent hepatic steatosis accounts for an independent association between NAFLD and incident T2D [62].

 Individuals with NASH are more likely to develop incident T2D during a median 3.5-year follow-up [63].

 NAFLD regression (compared with persistent NAFLD) is associated with decreased risk of incident T2D only in NAFLD patients with low NFS [64].

 The *PNPLA3* GG genotype aggravates liver steatosis associated with changes in body weight while reducing the risk of incident T2D in individuals with MUO [65].

 PPARγ agonists, such as pioglitazone, can induce NASH remission in patients with noncirrhotic NASH, regardless of the presence or absence of T2D, indirectly proving the pathogenic role of IR in NASH [47].

 Abbreviations. HCC—hepatocellular carcinoma; IR—insulin resistance; LT—liver transplantation, NAFLD—nonalcoholic fatty liver disease; NASH—nonalcoholic steatohepatitis; NFS—NAFLD fibrosis score; T2D—type 2 diabetes; MUO—metabolically unhealthy obesity.

### 2.2. NASH and Obesity

NASH is strongly associated with obesity. Excess adiposity is related to dysfunctional adipose tissue and altered adipokine secretion [66]. This leads to increased lipolysis rates and higher free fatty acid release from adipose tissue, leading to ectopic fat accumulation in different tissues, including the liver [67]. The relationship between expanded/dysfunctional adipose tissue and liver disease appears to be present at the early stages of the disease, even when cardiometabolic risk factors have not fully developed [68]. For example, a prospective French study conducted at a tertiary referral center on a cohort of 837 individuals who had undergone liver biopsy during bariatric surgery found that NASH was independently associated with larger waist circumference and that even among those with metabolically healthy obesity (MHO), 5.4% had NASH and 24.6% had liver fibrosis ≥ F2 [69]. A smaller-sized study conducted among 141 German patients undergoing bariatric surgery found that an increasing proportion of definite NASH cases could be seen in parallel with increasing numbers of criteria of metabolically unhealthy obesity (MUO), and a higher homeostasis model assessment of insulin resistance (HOMA-IR) score was the only independent predictor of NASH [70], again suggesting that insulin resistance is the driver for all metabolic abnormalities and, at least in part, the development and progression of liver disease. NASH and T2D predict poor weight loss after sleeve gastrectomy [71]. Interestingly, the most accurate fibrosis biomarkers among individuals with obesity are those that are unaffected by BMI, such as the FIB-4 index and PRO-C3-based testing [72]. From a molecular perspective, it is important to note that, given the dissociation between STAT-1 and STAT-3 activation pathways, obesity may contribute to the development of NASH and hepatocellular carcinoma (HCC) through different independent mechanisms. Grohmann et al. [73], examining mice models of obesity and NASH, found that obesity drives NASH via STAT-1 signaling while promoting HCC via STAT-3 signaling. Consistently, these researchers successfully showed that the attenuation of STAT-1 signaling prevents NASH (but not HCC), while inhibition of STAT-3 signaling blocks HCC development (but not NASH) [74]. This discovery probably accounts for the worrying clinical feature that HCC may develop in noncirrhotic NASH [75].

### 2.3. NASH and Atherogenic Dyslipidemia

NAFLD is typically associated with high plasma triglycerides and low HDL-cholesterol concentrations [76,77]. Initially, increased hepatic triglyceride accumulation results in compensatory over-secretion of VLDL particles with increased triglyceride content [78]. However, this compensation usually cannot match the lipids overflowing the liver in these patients [79]. The normal CETP-mediated exchange of triglycerides and cholesterol that occurs between lipoproteins is likely affected by the triglyceride-rich VLDL particles. This process culminates in the production of low HDL cholesterol and small, dense proatherogenic LDL particles [80]. These plasma lipoprotein abnormalities are driven by increased insulin resistance and hepatic steatosis rather than by the severity of liver histology [81]. However, advanced liver disease exhibits reduced protein synthesis, altered protein composition, and impaired function of these plasma lipoproteins [82].

### 2.4. NASH and Metabolic Syndrome

During approximately 20 years of investigations, many studies independently conducted by different researchers have concluded that metabolic syndrome and NASH are intimately associated [68,83,84,85,86,87,88]. This notion is confirmed by some studies, analyzed below, which have also addressed “at-risk NASH”.

In a study of 118 consecutive biopsy-proven NAFLD cases, Ballestri et al. [18] found that the independent predictors of NASH were waist circumference, HOMA-IR, metabolic syndrome, and circulating levels of alanine aminotransferase (ALT), uric acid, and total cholesterol; the independent predictors of NAS ≥ 5 were serum ALT, uric acid, and total cholesterol levels; the independent predictors of significant and advanced fibrosis were age, waist circumference, metabolic syndrome, serum ALT, total cholesterol, platelet count, HOMA-IR, and T2D. For more-than-mild portal inflammation, the predictors were serum aspartate aminotransferase (AST), serum iron, NAS ≥ 5, and significant liver fibrosis.

Alexoupolos et al. [89] conducted a study correlating metabolic dysfunction and liver histology changes among 713 United States individuals with histologically confirmed NAFLD/NASH (49% of whom had T2D). These investigators showed that, compared with good glycemic control, moderate glycemic control was significantly associated with increased severity of ballooned hepatocytes (OR 1.74; 95% CI 1.01–3.01) and hepatic fibrosis (OR 4.59; 95% CI 2.33–9.06). The relevance of these results is both pathogenic and clinical, highlighting that poor glycemic control predicts the severity of a cardinal feature of NASH diagnosis (i.e., ballooned hepatocytes) and a prognostic factor of NASH (i.e., liver fibrosis). These results also suggest that optimizing glycemic control in people with T2D might reduce the risk of liver fibrosis progression. However, as mentioned previously, this is not a straightforward relationship. While plasma glucose concentration is used as a substrate for hepatic de novo lipogenesis (DNL) and there is evidence that higher glycosylated hemoglobin (HbA1c) in patients with T2D is associated with worse liver fibrosis, these associations are evident only in the presence of chronic hyperinsulinemia, which is a strong determinant of hepatic DNL [90]. Therefore, a fine-tuned equilibrium between overall plasma glucose and insulin levels may determine the impact on liver health. This might also partly explain the conflicting results observed after using insulin therapy in liver fat accumulation [50,91]. It can also explain why, despite the successful glucose-lowering effect, long-acting pegylated insulin with preferential action in the liver (as opposed to adipose tissue and skeletal muscle) resulted in higher liver fat accumulation and toxicity [92].

In a study of 376 medicolegal autopsies, Klaric et al. [93] reported that the prevalence of some metabolic syndrome features (i.e., T2D and BMI) and postmortem HbA1c levels were significantly higher in NASH cases than non-NASH cases. Decedents with moderate/severe liver fibrosis also had a higher prevalence of T2D, BMI, and HbA1c than those with no/mild fibrosis. Finally, a postmortem HbA1c ≥ 7% was found to be an independent predictor of NASH (OR 5.11, 95% CI 2.61–9.98) and advanced fibrosis (OR 3.94, 95% CI 1.63–9.53).

Using the National Health and Nutrition Examination Survey (NHANES) database, Payne et al. [94] provided data conceptually agreeing with the above findings, showing that the presence of metabolic comorbidities (i.e., T2D, obesity, metabolic syndrome, and insulin resistance) was associated with a 1.3 to 1.7 times greater prevalence of “at-risk NASH” (noninvasively identified by a FibroScan-AST (FAST) score > 0.35). Moreover, preexisting T2D, increased waist circumference, and low HDL-cholesterol levels independently predicted “at-risk-NASH”. The number of metabolic syndrome components was also important—one additional component increased the odds of at-risk NASH by two times.

### 2.5. Key Role of Metabolic Dysfunction in NAFLD/NASH Pathogenesis

**Figure 1:**
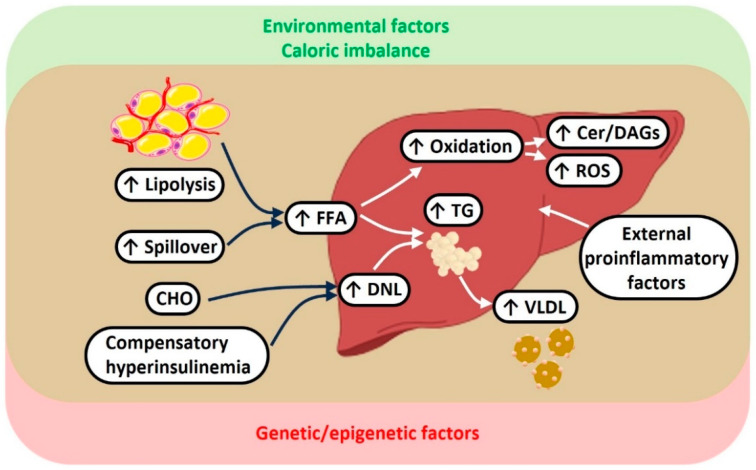
Multifactorial pathogenesis of NAFLD and its progression to NASH. A combination of environmental, inflammatory, genetic, and epigenetic factors promote the development of insulin resistance. Through different mechanisms, insulin resistance is closely associated with increased FFA influx to the liver and increased hepatic de novo lipogenesis. In turn, lipid excess leads to compensatory (and over-compensatory) responses, including hepatic triglyceride accumulation (steatosis), VLDL secretion (high plasma triglyceride levels), and increased hepatic fat oxidation with generation of inflammatory lipid intermediates and reactive oxygen species. Abbreviations: CHO: carbohydrates; FFA: free fatty acids; DNL: de novo lipogenesis; TG: triglycerides; VLDL: very low-density lipoproteins; Cer: ceramides; DAG: diacylglycerols; ROS: reactive oxygen species.

NAFLD has a complex and multifactorial pathogenesis involving increased influx to the liver of non-esterified fatty acids, driven by visceral adiposity and peripheral insulin resistance, together with increased DNL and impaired oxidation of fatty acids and export of VLDL particles into the bloodstream; intestinal dysbiosis and specific genetic polymorphisms may also contribute to increasing NAFLD risk [95]. In NASH, like in T2D [96], insulin resistance appears to be a “necessary, but not sufficient” player [97], which acts in concert with many disease modifiers of genetic, epigenetic, and environmental nature (including lifestyle factors, drugs, and viral infections) [12]. This complex backset offers a conceptual reading frame helping to explain why some, but not all, individuals with NAFLD ultimately develop NASH, and why only a subset of individuals with NASH progress to cirrhosis and end-stage liver failure over time [12]. The multifactorial pathogenesis of NASH in the setting of insulin resistance is schematically summarized in Figure 1.

NASH pathogenesis features the reprogramming of major cell types, such as Kupffer cells, hepatic stellate cells, hepatocytes, cholangiocytes, endothelial cells, leukocytes, macrophages, and intrahepatic T cells, which occurs during NASH progression owing to cell-specific transcriptomic, metabolic, and functional remodeling [98].

Varying degrees of exposure and responses to metabolic stresses, susceptibility to hepatocyte lipotoxicity, and differences in repair-response efficacy, as well as their interplay, likely account for the high heterogeneity of NASH (Table 1), thus strongly justifying personalized medicine approaches in this arena [99,100].

According to the so-called “lipotoxicity theory”, the occurrence of hepatocellular injury and lobular inflammation—which distinguish NASH from uncomplicated steatosis (NAFL)—can result from the chemical types of fatty substrates accumulated and how they are stored in intrahepatic lipid droplets (a phenomenon that is under control of genetic polymorphisms, such as the PNPLA3 I148M variant) rather than from the extent of intrahepatic fat accumulation [101]. The lipotoxicity theory highlights the possibility that, while triglycerides are chemically inert and do not have the property to damage the hepatocytes, other lipid substrates may trigger more complex and dangerous biological events, such as mitochondrial injury and endoplasmic reticulum (ER) stress, eventually culminating in activation of c-Jun N-terminal kinase (JNK) and release of danger-associated molecular patterns (DAMPs), apoptosis, necroptosis, pyroptosis, or autophagy [101,102,103].

The circadian clock machinery also controls metabolic homeostasis in the liver and participates in ER stress-related pathways, thus playing a role in the development of NASH [104]. In their turn, DAMPs stimulate innate immunity by binding pattern recognition receptors, such as toll-like receptor 4 (TLR4) and the NOD-like receptor protein 3 (NLRP3) inflammasome, with ensuing release of the inflammatory cascade sustained by chemokines and cytokines (interleukin (IL)-1β and IL-18) to attract inflammatory cells (e.g., activated macrophages), which typically surround ballooned hepatocytes as crown-like structures. Injured cell-derived extracellular vesicles that circulate in the bloodstream of NASH patients may also contribute to the inflammatory state of this liver disease and set the stage for hepatic fibrogenesis and, in a proportion of cases, hepatocarcinogenesis [101]. Oxidative stress is a key driver of NASH progression through the transcription factor NRF2 and its negative regulator KEAP1, which are candidate targets for NASH drug management [105].

In individuals with metabolic dysfunction, liver damage triggers the coagulation cascade, which plays a key role in both hepatic fibrogenesis and histogenesis of NASH-related cirrhosis [40,106].

The chemically reactive, hence hepatoxic, lipid species involved in NASH pathogenesis include free (unesterified) cholesterol crystals, saturated free fatty acids (conversely unsaturated fatty acids are protective), diacylglycerols, lysophosphatidylcholine, sphingolipids, and ceramides [101]. Interestingly, lipidomic alterations featured by NASH, including elevated saturated and monounsaturated hepatic lipids and a reduced proportion of polyunsaturated hepatic lipids, are directly associated with metabolic abnormalities, such as insulin resistance [107].

Further to hepatocytes, other cell types, including hepatic stellate cells and Kupffer cells, may play a major role in the fibrotic progression of NASH [95], and inflammatory stimuli in NASH also typically result from extrahepatic sites, such as adipose tissue, skeletal muscle, and intestine [108].

Illustrating the notion that genetic and metabolic factors synergistically interact in the development and fibrotic progression of NASH, a study from Japan reported that individuals with metabolic and genetic risk factors had a markedly increased risk for NASH, significant fibrosis, or advanced fibrosis (OR 12.30, 5.50, and 6.25, respectively) [109].

An adverse metabolic milieu affects the immune response, which plays a crucial role in the pathogenesis of NASH. For example, the functional status (“polarization”) of macrophages is influenced by fatty acids, which, in turn, promote the progression of metabolic dysfunction across various phases of disease and in different body tissues [110,111,112]. Macrophages play a crucial role in liver fibrosis progression and reversal via IL-4Rα signaling [113]. Moreover, the immune system can contribute to all histological stages of NAFLD, and other immune cell types may also be involved across the NAFLD/NASH spectrum (auto-aggressive T cells, unconventional T cells, and platelet-immune cell interactions) in close association with metabolic dysregulation [114]. Notably, CD8+ tissue-resident memory cells play a crucial role in resolving liver fibrosis by inducing apoptosis of hepatic stellate cells [115].

Recent studies in the NASH research field addressed the pathogenic role of low-grade inflammation and vascular impairment, perturbed immunometabolism, and a prothrombotic milieu, finally culminating in profibrogenic hepatic hypoxia [116]. In this context, Arelaki et al. investigated neutrophil extracellular traps (NETs), acknowledged to be involved in the pathogenesis of various noninfectious proinflammatory and prothrombotic conditions, in liver biopsies of 20 individuals with NASH [117]. Data have shown that NETs were common (occurring in approximately 95% of the biopsy specimens) and specific in NASH (absent in normal and diseased control specimens). Additionally, NETs were significantly (*p* < 0.001) associated with the presence of NASH and all its individual histological features (steatosis, ballooning degeneration, lobular inflammation and portal inflammation), and were garnished with IL-1β and IL-17A. Moreover, platelet aggregates were much larger in NASH specimens than in control specimens. Collectively, these data support a role for thromboinflammation in the pathogenesis of NASH [117]. It is appreciated that the immune-mediated response in NASH involves both adipocytokines and increased intestinal permeability owing to gut dysbiosis; translocation of bacterial endotoxins will ensue, initially triggering the intrahepatic proinflammatory cascade of toll-like receptor 4 (TLR4) in the liver, and eventually culminating in the activation of hepatic stellate cells and signals converging toward a fibrotic phenotype owing to the chronic inflammatory activation of the liver-resident cells, as well as the necrotic and apoptotic pathways [118].

Sex-related differences are identifiable in NASH pathobiology in humans and experimental models across various physiological functions spanning hepatic steatogenesis to immune response and fibrogenesis [28]. Sex hormones regulate body fat distribution, with women typically accumulating gluteofemoral adipose tissue and men being prone to visceral adiposity. Moreover, muscle physiology, sarcopenia, and responses to treatment are also different between men and women. Finally, hepatic insulin resistance is higher in men than in BMI-matched women. Taken collectively, these sex-related differences fully support the recommendations to conduct NASH therapeutic trials, taking sex and reproductive status into adequate consideration [28].

Collectively, the above studies fully document the notion that NASH is a systemic metabolic disorder, the initiation and worsening of which is facilitated by genetic predisposition and modulated by sex and reproductive status. Moreover, confirming the pathogenic importance of metabolic risk factors in NASH, experimental data using dietary interventions with either valine or isoleucine corrected liver diacylglycerols and gene expression of multiple metabolic processes via hepatoprotective effects on oxidative stress and inflammatory proteins and improved NASH histology features in a genetically engineered mouse model [119].

## 3. Pitfalls in Endpoints in NASH Clinical Trials

Several conceptual weaker points can be identified in currently adopted endpoints in NASH trials.

### 3.1. Surrogate Outcomes May Be Dissociated from Hard Clinical Outcomes

In 2019, Adams was the first to pinpoint that while the current drug targets are primarily directed at improving liver histology outcomes (steatosis, injury, and fibrosis), the leading cause of mortality among NAFLD patients is cardiovascular disease (CVD), and so, ideally, an effective pharmacological treatment should address CVD risk factors and mortality rather than a reduction in liver-related mortality [120]. Additionally, given that the rate of liver-related mortality is as low as 1.7% over 7 years among community-based NAFLD patients and 1.7% yearly among patients with compensated NASH-related cirrhosis [121,122], it has been calculated that thousands of patients should be followed over many years to show efficacy for the endpoint of liver-related mortality [120]. Given that it heralds a mortality risk of around 50% at 2 years among patients with NAFLD [123], hepatic decompensation is a clinically relevant endpoint. However, it also remains logistically challenging due to its low rate of development [120]. These considerations fully illustrate the logistical difficulties of demonstrating drug efficacy on hard liver-related clinical endpoints, such as hepatic decompensation or liver-related death, accounting for the logic of surrogate markers of these endpoints being accepted by regulatory authorities, including the Food and Drug Administration (FDA) and European Medical Agency (EMA), for the conditional approval of a new drug for NASH [120]. However, it should be kept in mind that surrogate liver-related outcomes may be misleading if off-target drug effects lead to adverse patient outcomes despite an improvement in the surrogate endpoint(s) [124].

### 3.2. Limitations of Liver Biopsy and Histologic Assessment

Although liver biopsy is currently the gold standard for diagnosing NASH, it has many drawbacks (as summarized below).

First, liver biopsy is an invasive diagnostic procedure not without risks and possible acute complications. A recent meta-analysis of 30 studies reporting complications rates from 64,356 percutaneous liver biopsies identified major complications in ~2.5% of cases, with a mortality rate of 0.01%, a hospitalization rate of 0.65%, major bleeding in ~0.5% of cases, and moderate/severe pain in ~0.35% of cases [125]. Interestingly, although patients considered it a helpful procedure, and they widely accepted undergoing a percutaneous liver biopsy, one out of six individuals would have instead chosen a less aggressive diagnostic technique (even if it provided less information). Moreover, some patients highlighted that liver biopsy did not involve therapeutic changes in about 50% of cases [126]. Twenty years ago, some researchers envisaged terms such as “power, oppression and violence” to describe liver biopsy [127]. Hesitancy to accept liver biopsy is a limitation in recruiting patients to enroll in phase 3 NASH clinical trials [128].

Second, given that the NASH histology changes are not uniformly distributed within the liver, sampling errors and inter-rater variability can occur, thus limiting the use of this invasive procedure in clinical practice [129]. Ratziu et al. conducted a seminal study investigating 51 patients with NAFLD who underwent liver biopsy with simultaneous sampling of two specimens [130]. These authors reported that histological lesions of NASH were unevenly distributed throughout the two liver specimens. As a result, sampling errors of liver biopsy can lead to substantial NASH misdiagnosis and fibrosis staging inaccuracies.

Third, the agreement among various liver pathologists is incomplete, especially in identifying hepatocyte ballooning, lobular inflammation, and diagnosis of NASH [131,132].

Fourth, coming to the targets requested by the FDA, liver biopsy results are not reliable treatment response measures, given that the interobserver weighted kappa scores for the two intermediary approvable histological endpoints in NASH trials were poor: 0.396 for NASH resolution without worsening of fibrosis and 0.366 for ≥1 stage fibrosis improvement without worsening of NASH, respectively [34].

Fifth, the lack of standardization across NASH clinical trials has been highlighted regarding several aspects spanning from the number of readers, consensus between these, number of slides to be read, digital versus glass slide reads, to the possibility of rereading baseline slides [133].

Sixth, the surrogate liver-related outcomes requested by regulatory authorities exhibit spontaneous fluctuations. Evidence for this has come from analysis of the placebo responses in NASH clinical trials showing that, among patients randomly assigned to placebo, hepatic steatosis improved by up to ~33%, hepatocyte ballooning by ~30%, lobular inflammation by ~32%, and fibrosis stage by ~20% [134]. Predictors of a higher placebo response include those NASH clinical trials with higher baseline NAS conducted in South America and those in which patients had weight loss [135]. All these items should be given consideration when interpreting and designing future NASH therapeutic trials.

Seventh, the surrogate liver-related outcomes may be internally inconsistent, given that the severity of NASH and the severity of fibrosis are two different, distinct, and possibly independent concepts with different classifications. Moreover, staging of fibrosis severity with numerical descriptors may be misleading given that the absolute amount of hepatic fibrosis increases nonlinearly (but exponentially) with each numerical stage [136]. For example, the severity of steatosis, inflammation, and hepatocellular ballooning, which may be conceptualized as correlates of metabolic disease activity, are a recognized driver of liver fibrosis, which best mirrors the severity of NASH [18,136,137]. Moreover, confirming the notion that different stages of liver fibrosis may confer an exponential (not an additive) risk of adverse liver outcomes, patients with NASH and fibrosis stage F3 have a ~five-fold higher risk of liver-related events than those with no or mild liver fibrosis [138].

In the TARGET-NASH study conducted on a cohort of 3474 middle-aged subjects to determine patient factors predictive of histologic versus empiric clinical diagnosis of NAFLD in real-world practice, Barritt et al. reported that about two thirds of patients with NAFLD did not have a liver biopsy [139]. These non-biopsied patients were more likely to be non-White, older, and with a normal serum ALT concentration, showing potential gaps in our knowledge about this population.

### 3.3. Noninvasive Tests for the Diagnosis of NASH Are Far from Perfect

The above notions extensively illustrate the urgent need for accurate, noninvasive tests (NITs) to diagnose NASH. Blood-based biomarkers (“wet test”) and imaging techniques (“dry tests”) have been recently discussed in comprehensive systematic reviews [140,141,142,143,144]. An increasing number of NITs have been developed for the diagnosis of NAFLD, definite NASH, or at-risk NASH, as well as clinically significant or advanced fibrosis. Some new imaging methods (e.g., magnetic resonance imaging (MRI)-PDFF, MR spectroscopy, and MR elastography) have shown promising results and are frequently used as primary outcomes in phase 2 clinical trials. However, there are conflicting data regarding whether longitudinal changes in these imaging techniques can accurately predict changes in liver biopsies [145,146]. Moreover, these imaging methods are not widely available and expensive, two important factors limiting their widespread use in large phase 3 clinical trials. Blood-based biomarkers and/or clinical scores have shown poor results in predicting histological liver outcomes. Despite significant efforts, no NIT has been able to diagnose NASH and predict accurately its resolution. Due to all these limitations, the FDA only supports the use of NITs for either early drug development or for screening purposes in larger studies to select patients for a liver biopsy [147].

### 3.4. Large Variability in Placebo Responses among Histological Outcome Trials

For multiple reasons, in NASH therapeutic trials, placebo groups have shown highly variable histological responses [135]. For example, improvement in one stage of liver fibrosis without worsening of NASH was achieved by 12% of patients in the REGENERATE trial [148] and was almost three times higher (e.g., 33%) in the semaglutide trial [55]. Consequently, while liver biopsy reading variability is likely an important factor, as described above, other factors should also be considered. Among these, it is important to mention the duration of therapy, the intensity of lifestyle intervention performed in the placebo group, the final weight loss achieved, and the basal severity of liver disease, among others. Whether other factors, such as the patient’s age, duration of liver disease, changes in macronutrient composition, etc., also play a role in the variability observed remains to be determined.

## 4. Why Do NASH Clinical Trials Fail?

**Figure 2:**
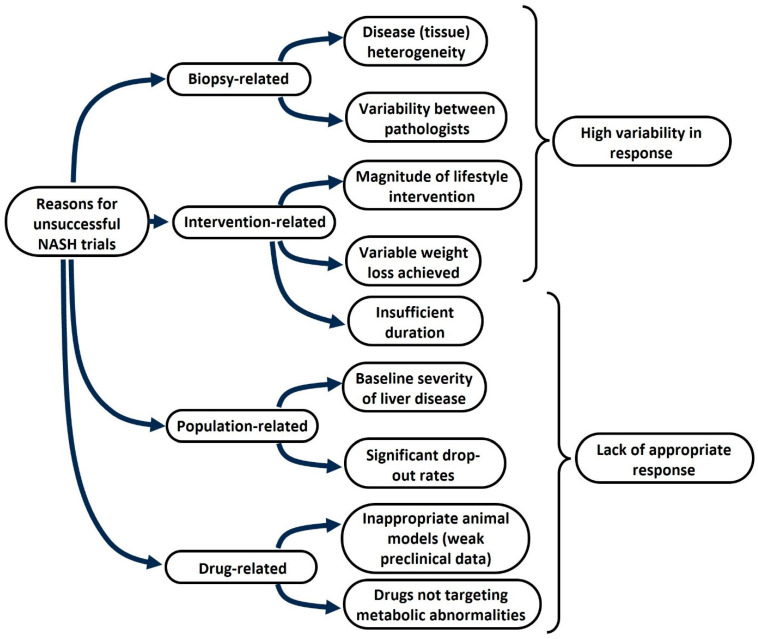
Multiple reasons may explain the high rate of unsuccessful phase 2 and phase 3 NASH clinical trials. The lack of success in phase 2 and phase 3 clinical trials in patients with NASH ± liver fibrosis can be explained by multiple factors, which can be schematically divided into biopsy-related, intervention-related, population-related, and drug-related factors.

Experts have been interrogating themselves on the likely reasons underlying the failure of NASH clinical trials for almost a decade (as summarized in Figure 2). Various explanations have been recognized over time, including patient population heterogeneity, slow disease progression, and requiring liver biopsies to diagnose and assess liver fibrosis and treatment response [123]. Additionally, studies have identified issues in the recruitment of patients, efficacy of both drugs and placebos, stratification of enrolled patients for lifestyle habits, and metabolic comorbidity at the baseline as well as during the progression from phase 2 to phase 3 clinical trials [149,150]. As reported by Ratziu et al. [151], various preclinical and clinical considerations may impact clinical trial outcomes. The former include experimental disease models in animals carrying little resemblance to human NASH, for example, owing to higher reversibility of liver fibrosis than in humans who generally have longer disease duration; incomplete understanding of target biology and disease pathogenesis or drugs insufficiently modulating biological targets; existence of redundant physiological mechanisms able to compensate for drug-induced target modulation; efficacy of the drug candidate not sufficiently supported by a variety of available techniques (such as genetic inactivation, pharmacological inhibition, antisense oligonucleotides, or small interfering RNA); insufficient extent of/inconsistent antifibrotic effect; or antifibrotic effect dissociated from pathways of liver injury, inflammation, or cell death. The latter comprise absent/insufficient proof-of-concept demonstration provided by preliminary trials; phase 2 trials biased with a high rate of false discovery; overinflated effect size; failure to show any efficacy on secondary endpoints; underpowered studies; utilization of suboptimal drug doses and uncertain response rate due to placebo; changes in patient population from phase 2 to phase 3; alcohol intake; and metabolic worsening occurring during the trials. For NASH-related fibrosis trials, they comprise unreliable noninvasive assessment of changes in liver fibrosis or uncertain duration of the study to show any fibrosis improvement owing to indirect antifibrotic mechanisms. For NASH-related cirrhosis trials, they comprise the following: stage of disease too advanced; or the existence of a multiplicity of ongoing pathogenic processes (e.g., portal hypertension, bacterial translocation, and sepsis), which cannot be controlled by antifibrotic drugs alone.

Tilg et al. [47] raised concerns related to difficulty in identifying drug-induced liver injury (DILI), which is potentially associated with acute liver failure and mortality. Indeed, DILI diagnosis requires a liver biopsy to articulate a differential diagnosis. Moreover, these authors highlighted that, given the systemic nature of NAFLD/NASH, in which cardiovascular events are the leading cause of mortality, noninvasive biomarkers of cardiovascular disease are urgently needed, and cardiometabolic safety is a requirement for NASH drug candidates. Additionally, attention is attracted to the duration of clinical trials given that regression of (advanced and cirrhotic) liver fibrosis predictably takes years to occur. Furthermore, patient phenotypes vary considerably, and we do not have a one-size-fits-all drug. A range of treatment options exists for NAFLD, but liver efficacy endpoints are not the only necessity when faced with a multisystem disease affecting multiple extrahepatic organs.

## 5. Can We Extrapolate Results from Cardiovascular Outcome Studies in Obesity and T2D to Patients with NAFLD or NASH?

A 2007 meta-analysis suggesting that rosiglitazone was associated with a significant increase in the risk of acute myocardial infarction [152] resulted in a new 2008 FDA recommendation for the need for long-term cardiovascular safety trials for new glucose-lowering drugs. The implementation of these new large trials resulted in the discovery that several drugs initially developed due to their glucose-lowering effects had additional benefits on cardiovascular events and mortality [153,154,155], heart failure [156,157,158], and renal outcomes [159,160] in patients with T2D. Of note, not only studies on heart failure and kidney disease that included patients without diabetes but also post hoc analyses from large cardiovascular outcome trials showed that the improvement in cardiovascular events was independent of glycemic control [161]. More recently, in the SELECT trial (enrolling 17,604 adult patients randomly assigned to receive subcutaneous semaglutide or placebo), Lincoff et al. [162] reported that in patients with preexisting cardiovascular disease and overweight or obesity but without T2D, weekly subcutaneous semaglutide at a dose of 2.4 mg was superior to the placebo in reducing the incidence of death from cardiovascular causes, nonfatal myocardial infarction, or nonfatal stroke (hazard ratio 0.80; 95% CI 0.72–0.90) at a mean follow-up of 39.8 months. Because patients with overweight, obesity, or T2D represent the large majority of those with NAFLD, these results are likely to extrapolate to most patients with NAFLD. It is also likely that these results may translate to patients with NASH and even advanced fibrosis, based on the metabolic benefits observed in these patients with a glucagon-like peptide 1 (GLP-1) receptor agonist, such as subcutaneous semaglutide [55,163]. Recent hepatological and endocrinological guidelines recommended initiating pioglitazone and/or GLP-1 receptor agonists, especially in patients with biopsy-proven NASH and T2D [164,165]. Considering this, should we rearrange the NAFLD therapeutic landscape? Are we focusing too much on liver histology outcomes and forgetting about much more pressing issues, such as overall mortality and cardiovascular disease? Our response is probably yes.

## 6. Research Agenda

NASH is a major clinical and public health issue that is intimately associated with metabolic dysfunction, often in the context of obesity, T2D, and metabolic syndrome. Contrasting with this systemic backset, the surrogate histological endpoints in NASH clinical trials established by the American and European regulatory agencies fail to prioritize a holistic approach, including metabolic dysregulation and cardiovascular risk, and instead focus on liver histology outcomes. Unsurprisingly, most NASH clinical trials have yielded quite disappointing results so far, and the lack of approved pharmacotherapies for patients with NASH represents a substantial unmet medical need [166].

The notable placebo response rate identified in early phase studies of NASH mirrors the lack of standardized approaches to lifestyle recommendations, renders cross-trial comparisons impracticable, and hinders the assessment of novel therapeutics [167]. However, the rates of liver fibrosis progression and regression are much higher in randomized clinical trials than in observational studies [168], calling for studies to bridge the gap between clinical practice and sponsored research.

Even though biological sex is a significant determinant of health, disease, and medicine, and sex dimorphism is a definite feature of NAFLD and NASH, most of clinical trials evaluating new drugs for NAFLD fail to report any data on sex-specific response rates and sex-related outcomes [169,170]. Well-characterized hormonal changes may occur with aging, implying that not only sex but also age and reproductive status, which may affect the clinical presentation and course of NAFLD/NASH, should receive full consideration through sex- and age-specific analyses of clinical trials [171]. This would avoid the well-known phenomenon of women being generally disadvantaged in recruitment in clinical trials owing to pregnancy-related safety concerns and drug companies being interested in setting the most favorable and easy conditions to test drug safety and efficacy [171].

Although NASH is defined histologically, liver biopsy poses various challenges impacting patient enrollment, observers’ agreement, and extension to the clinical practice of trial findings [172]. Moreover, the intrinsic imprecision of liver biopsy makes currently adopted histological endpoints in NASH clinical trials both fallacious and overambitious and strongly advises in favor of identifying noninvasive biomarkers and criteria for entry into NASH trials and evaluating drug efficacy [34]. Moreover, follow-up liver biopsies for identification of post-treatment outcomes are rarely acceptable for patients and are not entirely free from risks.

Several serologic, imaging, and composite noninvasive tests are available or under development to provide an alternative surrogate for the histological assessment of steatosis, fibrosis, steatohepatitis, or metabolic dysfunction [173,174,175]. However, these surrogate biomarkers/tests are not currently accepted for the conditional approval of NASH drug candidates [176]. Ideal biomarkers are not yet available, and investigators should possibly look outside of the liver. For example, muscle fatty infiltration (perhaps a pathophysiological contributor) could be a potential marker for NASH [177].

Consideration of NASH as a systemic metabolic disease implies that any attempt to cure it is destined to fail unless weight loss and systemic/hepatic insulin resistance are contextually targeted. Accordingly, future NASH clinical trials should better stratify the metabolic comorbidities of participants [150]. Case definitions, standardized techniques of evaluation, and treatment endpoints require partnerships between all stakeholders involved to develop a consensus on adequate NASH endpoints that cannot be limited to hepatic effects but must also include cardiovascular endpoints, requesting a description of the initial cardiometabolic risk profile and appropriate adjudication of adverse events. To achieve these aims, the utilization of surrogate outcomes common to both liver and cardiometabolic diseases should be evaluated [178], and close collaboration between cardiologists, endocrinologists, and hepatologists is required [179].

Two studies have provided excellent examples of a holistic approach. Lin et al. showed that ezetimibe (compared to a placebo) significantly improved the Framingham risk score [180], a validated sex-specific algorithm used to estimate the individual 10-year cardiovascular risk [181]. Ratziu et al. [182] reported that vonafexor (an FXR agonist under development for fibrosing NASH) not only induced a significant reduction in body weight, hepatic steatosis, and serum liver enzyme levels, but also improved renal function parameters. This is important given that patients with NAFLD are more likely also to develop chronic kidney disease [183,184].

Evaluation of the economic benefits of NASH treatment and its impact on patients’ quality of life, extrahepatic manifestations, and consequences on public health is a major concern [162], and so are patients’ needs and patient-centered outcomes; funding and sustainability scenarios and the potential for the extension of adult treatments to children should all be kept in consideration [185,186].

Increasing patient homogeneity would permit more reliable comparisons of NASH treatment effects between studies. Consistently, more accurate phenotypic stratification of participants and greater uniformity of confounding variables may eventually lead to accelerated development of new drugs for NASH [187,188,189]. Finally, harmonizing the FDA and EMA guidelines is a research priority [189].

Addressing differences in treatment responses across different NASH clinical trials also requires a more standardized assessment of the physical activity level and ethnic, geographic, and genetic differences to gain additional insights into placebo response rates [167]. While the inadequate representation of women in NASH clinical trials remains a barrier to the implementation of evidence-based therapies [190], sex-related differences should be accurately addressed in the NASH research field owing to remarkable variations that occur in NAFLD pathobiology as a function of sex and reproductive status [28,191,192].

Deciphering which patients have more aggressive disease leading to fibrotic NASH remains an unresolved challenge. These individuals with “at-risk NASH” should be followed with surrogate markers and algorithms predicting both liver fibrosis regression and progression to cirrhosis and other clinically relevant liver-related and cardiovascular outcomes [193]. Artificial intelligence technology might also help, and machine learning algorithms accurately predict the hepatic venous pressure gradient (HVPG), clinically significant portal hypertension, and development of esophageal varices and HVPG changes in patients with NASH-related cirrhosis [194].

There exists a robust clinical rationale for developing drugs to treat NASH from hepatic and cardiometabolic approaches [47]. However, the multiplicity of clinically relevant outcomes associated with the complexity of NASH pathogenesis makes it unlikely that a single drug will reverse NASH. Therefore, combination pharmacotherapies for NASH have been advocated to improve efficacy and safety [195]. Although poorly defined, this drug composition should comprise drugs targeting the metabolic drivers of the disease offered to most NASH patients, some requiring hepatic anti-inflammatory and antifibrotic actions [196].

## 7. Conclusions

Data analyzed in our review concur in indicating that a “one size fits all” strategy is unlikely to succeed in NASH treatment, calling for precision medicine approaches [47]. To this end, the identification of genetic polymorphisms associated with response to specific drugs (e.g., obeticholic acid [197] and pioglitazone [198]) is a winning paradigm related to the evaluation of novel biomarkers easily applicable to real-world practice [195].

Advanced liver fibrosis may persist irrespective of weight loss [199], and treatment of NASH-related cirrhosis remains challenging. However, even a modest beneficial effect on liver fibrosis remodeling may be sufficient, with pioglitazone and subcutaneous semaglutide, for example, reducing liver fibrosis progression compared to placebo [55,186,200]. Optimal pharmacotherapies for NASH-related cirrhosis will have to address clinically meaningful and accessible outcomes [201]. Although not (yet) accepted by regulatory authorities, stabilization of early disease at risk of progression may reduce portal hypertension, allow endogenous hepatic regeneration, and improve quality and expectancy of life [201,202].

Alarmingly, most patients with NAFLD/NASH are unaware of their liver disease, highlighting the importance of increasing disease awareness with education campaigns [203,204] aiming at promoting healthy lifestyle habits and aggressive management of cardiometabolic comorbidities [205].

While effective NASH pharmacotherapies are predicted to be identified thanks to the refinement of trial endpoints and engagement of all stakeholders [206], to avoid “the risk of being blind in one eye”, an unbiased and holistic approach to NASH drug management must constantly be guaranteed.

## Figures and Tables

**Table 1:** The multiplicity of factors contributing to the heterogeneity observed in patients with NASH.

Mechanisms	Mediators	Hepatic Effects
Mechanisms leading to increased fat influx to the liver	Adipose tissue insulin resistance with increased lipolysis	They result in hepatic steatosis
	Hyperinsulinemia leading to hepatic de novo lipogenesis	
	Environmental factors (e.g., diet) resulting in spillover of FFA from lipoproteins, or increased load of carbohydrates	
	Genetic factors affecting the above processes and/or liver fat storage	
Compensatory mechanisms as a consequence of increased fat availability	Increased secretion of triglyceride-rich particles (e.g., VLDL)	Able to compensate at early stages of the liver disease
	Increased FFA oxidation	
	Increased liver fat accumulation	
	Genetic factors affecting the above processes	
Mechanisms linking liver fat excess to development of inflammation	Incomplete FFA oxidation with the generation of lipid intermediates, such as DAGs and ceramides	They contribute to the development of NASH
	Other toxic lipid species, such as free (unesterified) cholesterol	
	Increased mitochondrial respiration with the generation of reactive oxygen species (ROS)	
	Genetic factors affecting lipid storage	
Other mechanisms that contribute to NASH development or perpetuation	Genetic and epigenetic factors, including differences in sex and race	They contribute to the heterogeneity of NASH
	Immunological and vascular factors	
	Inflammatory and endothelial factors	
	Differences in repair-response efficacy	
	Mitochondrial injury and/or endoplasmic reticulum (ER) stress	
	Alterations in autophagy	
	Intestinal dysbiosis	

## Data Availability

This is a narrative review article where no new data were created.
